# Supplementary material for: Non-canonical reader modules of BAZ1A promote recovery from DNA damage
Source: Nat Commun. 2017 Oct 11;8:862. doi: 10.1038/s41467-017-00866-0 (PMC5636791; doi:10.1038/s41467-017-00866-0)
Supplement: Supplementary file 1 — Supplementary Information [file 41467_2017_866_MOESM1_ESM.pdf]

Description of Supplementary Files

File Name: Supplementary Information
Description: Supplementary Figures and Supplementary References

File Name: Supplementary Data 1
Description: RNA sequencing results from a 4-way comparison of re-expressing BAZ1A wild-type and BAZ1AKAKY mutant cells in control and UVC-irradiated conditions. Gene sets listed here are graphically represented in Fig. 7g. Significance cut-off is $p < 0.01$ and absolute log2 fold-change (FC) > 1. Enrichment for signaling pathways in the in "UP in UVC only" group was derived from the Reactome database (see the Methods section for more details).

File Name: Peer Review File

a

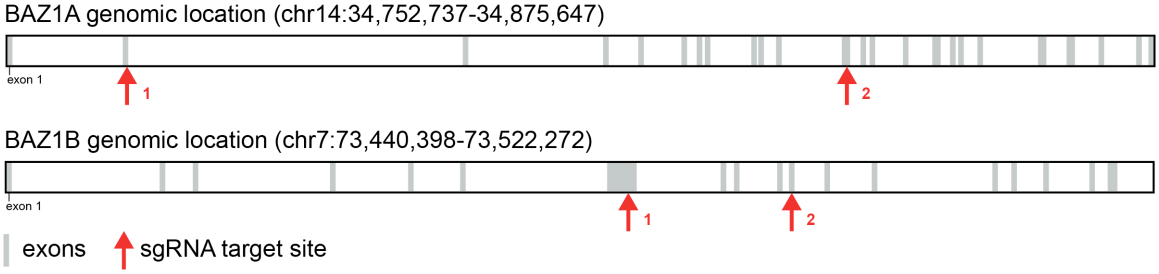

b

gene_sgRNA ID	19-mer targeting segment + 3-mer PAM
BAZ1A_sgRNA1	tcatgtaagcgcgaacgat ggg
BAZ1A_sgRNA2	gactgcacatcttagcttc agg
BAZ1B_sgRNA1	aaccttccagcattcagat tgg
BAZ1B_sgRNA2	actttctcttattccctga ggg

c

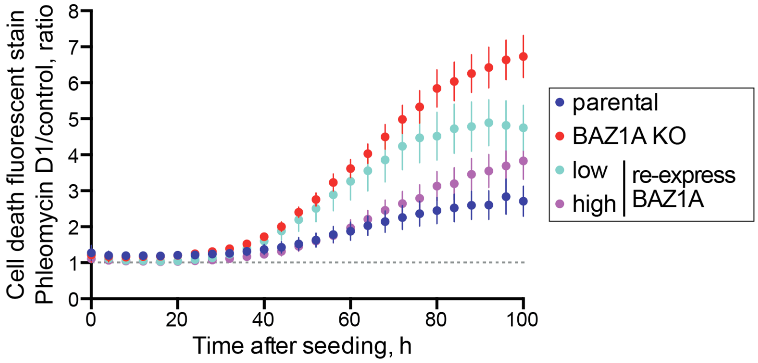

Supplementary Figure 1: Genome editing of *BAZ1A*- and *BAZ1B*-KO cells. (**a**) Schematic representation of the genomic loci for *BAZ1A* and *BAZ1B*. Exons and introns are shown in grey and white, respectively. Red arrows indicate the predicted target region of small guide RNAs (sgRNA) used for CRISPR-Cas9-mediated knockout cell line generation. (**b**) Sequences of sgRNA used for CRISPR-Cas9-mediated genome editing. (**c**) Cell death (green fluorescence) was measured over time in absence or presence of phleomycin D1. Rescue of DNA damage hypersensitivity of *BAZ1A* KO by “low” and “high” levels of BAZ1A expression. The grey dotted line indicates a ratio of 1, corresponding to the same level of cell death in absence or presence of phleomycin D1. Each time point shown is the mean value ± s.e.m from three independent experiments.

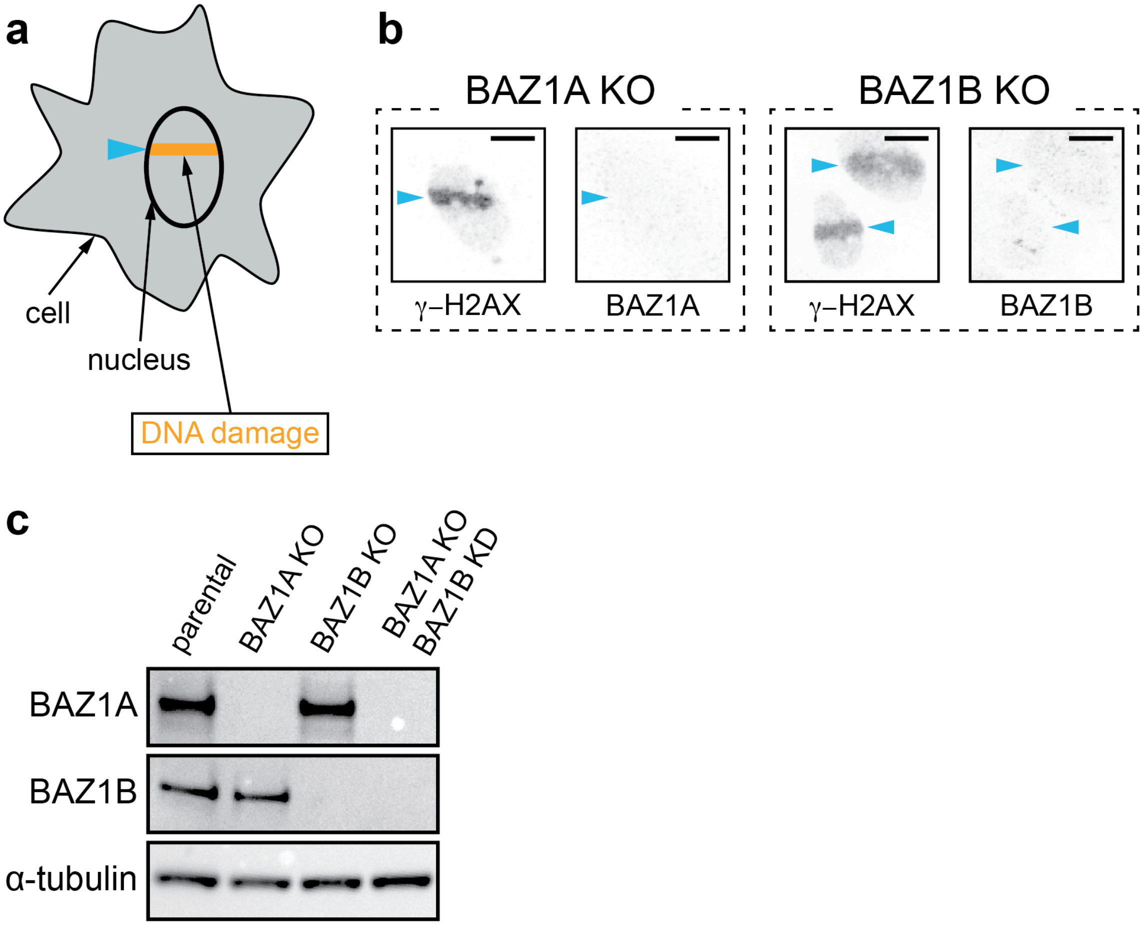

Supplementary Figure 2: CRISPR-Cas9 and RNAi-mediated depletion of BAZ1A and BAZ1B to monitor SMARCA5 recruitment to sites of DNA damage. (**a**) Schematic representation of a cell subjected to laser micro-irradiation. (**b**) Accumulation of BAZ1A and BAZ1B at sites of laser micro-irradiation in genome edited *BAZ1A*- and *BAZ1B*-KO cells, respectively. Signal for BAZ1A or BAZ1B is reduced to background when expression of the corresponding gene was abrogated by genome editing. Scale bar, 10 μm. (**c**) Western blot analysis of BAZ1A and BAZ1B expression in parental, *BAZ1A*-KO, *BAZ1B*-KO, and *BAZ1A*-KO cells depleted of BAZ1B by RNA interference (BAZ1A KO, BAZ1B KD).

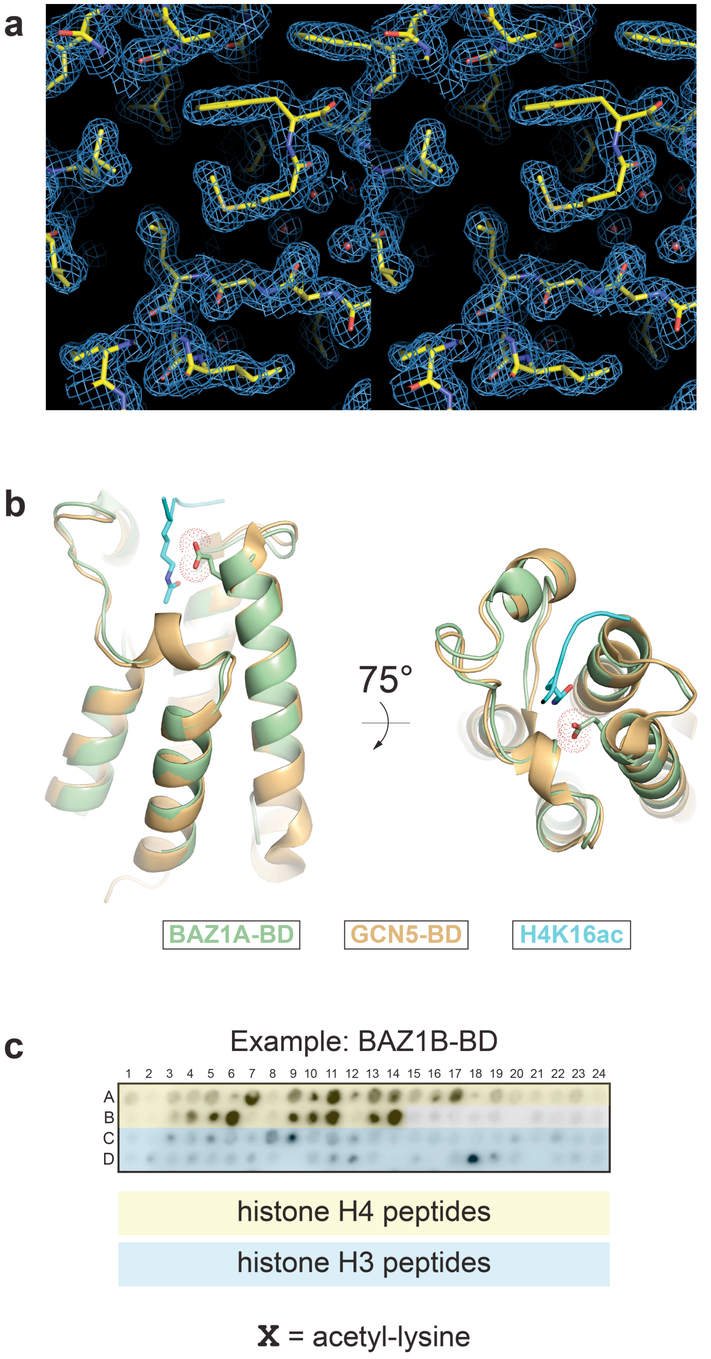

d

position	sequence	region
A 1	SGRGKGGKGLGKGGAKRHRKVLR	H4 1-23
A 2	SGRG**X**GGKGLGKGGAKRHRKVLR	H4 1-23
A 3	SGRGKGG**X**GLGKGGAKRHRKVLR	H4 1-23
A 4	SGRGKGGKGLG**X**GGAKRHRKVLR	H4 1-23
A 5	SGRGKGGKGLGKGGA**X**RHRKVLR	H4 1-23
A 6	SGRGKGGKGLGKGGAKRHR**X**VLR	H4 1-23
A 7	SGRG**X**GG**X**GLG**X**GGA**X**RHR**X**VLR	H4 1-23
A 8	SGRG**X**GG**X**GLGKGGAKRHRKVLR	H4 1-23
A 9	SGRGKGG**X**GLG**X**GGAKRHRKVLR	H4 1-23
A10	SGRGKGGKGLG**X**GGA**X**RHRKVLR	H4 1-23
A11	SGRGKGGKGLGKGGA**X**RHR**X**VLR	H4 1-23
A12	SGRG**X**GGKGLG**X**GGAKRHRKVLR	H4 1-23
A13	SGRGKGG**X**GLGKGGA**X**RHRKVLR	H4 1-23
A14	SGRGKGGKGLG**X**GGAKRHR**X**VLR	H4 1-23
A15	SGRG**X**GGKGLGKGGA**X**RHRKVLR	H4 1-23
A16	SGRGKGG**X**GLGKGGAKRHR**X**VLR	H4 1-23
A17	SGRG**X**GGKGLGKGGAKRHR**X**VLR	H4 1-23
A18	SGRGKGGKG	H4 1-9
A19	SGRG**X**GGKG	H4 1-9
A20	SGRG**X**GG**X**G	H4 1-9
A21	GKGGKGLGKG	H4 4-13
A22	GKGG**X**GLGKG	H4 4-13
A23	G**X**GG**X**GLGKG	H4 4-13
A24	GKGG**X**GLG**X**G	H4 4-13
B 1	G**X**GG**X**GLG**X**G	H4 4-13
B 2	GKGLGKGGAKR	H4 7-17
B 3	GKGLG**X**GGAKR	H4 7-17
B 4	GKGLG**X**GGA**X**R	H4 7-17
B 5	G**X**GLG**X**GGAKR	H4 7-17
B 6	G**X**GLG**X**GGA**X**R	H4 7-17
B 7	GKGGAKRHRKV	H4 11-21
B 8	GKGGA**X**RHRKV	H4 11-21
B 9	G**X**GGA**X**RHRKV	H4 11-21
B10	GKGGA**X**RHR**X**V	H4 11-21
B11	G**X**GGA**X**RHR**X**V	H4 11-21
B12	AKRHRKVLRD	H4 15-24
B13	AKRHR**X**VLRD	H4 15-24
B14	A**X**RHR**X**VLRD	H4 15-24
B15	GGGGG	Gly controls
B16	GGKGG	Gly controls
B17	GG**X**GG	Gly controls
B18	GGGGGGGGG	Gly controls
B19	GGGGKGGGG	Gly controls
B20	GGGG**X**GGGG	Gly controls
B21	SHLKSKKGQSTSRHKKLMFKTEG	p53 367-389
B22	SHL**X**S**XX**GQSTSRH**XX**LMF**X**TEG	p53 367-389
B23	SHLKSK**X**GQSTSRHK**X**LMFKTEG	p53 367-389
B24	blank	blank
C 1	ARTKQTARKSTGGKAPRKQLAT	H3 1-22
C 2	ART**X**QTARKSTGGKAPRKQLAT	H3 1-22
C 3	ARTKQTAR**X**STGGKAPRKQLAT	H3 1-22
C 4	ARTKQTARKSTGG**X**APRKQLAT	H3 1-22
C 5	ARTKQTARKSTGGKAPR**X**QLAT	H3 1-22
C 6	ART**X**QTAR**X**STGG**X**APR**X**QLAT	H3 1-22
C 7	ART**X**QTAR**X**STGGKAPRKQLAT	H3 1-22
C 8	ARTKQTAR**X**STGG**X**APRKQLAT	H3 1-22
C 9	ARTKQTARKSTGG**X**APR**X**QLAT	H3 1-22
C10	ART**X**QTARKSTGG**X**APRKQLAT	H3 1-22
C11	ARTKQTAR**X**STGGKAPR**X**QLAT	H3 1-22
C12	ART**X**QTARKSTGGKAPR**X**QLAT	H3 1-22
C13	STGGKAPRKQLATKAARKSAPAT	H3 10-32
C14	STGG**X**APRKQLATKAARKSAPAT	H3 10-32
C15	STGGKAPR**X**QLATKAARKSAPAT	H3 10-32
C16	STGGKAPRKQLAT**X**AARKSAPAT	H3 10-32
C17	STGGKAPRKQLATKAAR**X**SAPAT	H3 10-32
C18	STGG**X**APR**X**QLAT**X**AAR**X**SAPAT	H3 10-32
C19	STGG**X**APR**X**QLATKAARKSAPAT	H3 10-32
C20	STGGKAPR**X**QLAT**X**AARKSAPAT	H3 10-32
C21	STGGKAPRKQLAT**X**AAR**X**SAPAT	H3 10-32
C22	STGG**X**APRKQLAT**X**AARKSAPAT	H3 10-32
C23	STGGKAPR**X**QLATKAAR**X**SAPAT	H3 10-32
C24	STGG**X**APRKQLATKAAR**X**SAPAT	H3 10-32
D 1	QLATKAARKSAPATGGVKKPHR	H3 19-40
D 2	QLAT**X**AARKSAPATGGVKKPHR	H3 19-40
D 3	QLATKAAR**X**SAPATGGVKKPHR	H3 19-40
D 4	QLATKAARKSAPATGGV**X**KPHR	H3 19-40
D 5	QLATKAARKSAPATGGVK**X**PHR	H3 19-40
D 6	QLAT**X**AAR**X**SAPATGGV**XX**PHR	H3 19-40
D 7	QLAT**X**AAR**X**SAPATGGVKKPHR	H3 19-40
D 8	QLATKAAR**X**SAPATGGV**X**KPHR	H3 19-40
D 9	QLATKAARKSAPATGGV**XX**PHR	H3 19-40
D10	QLAT**X**AARKSAPATGGV**X**KPHR	H3 19-40
D11	QLATKAAR**X**SAPATGGVK**X**PHR	H3 19-40
D12	QLAT**X**AARKSAPATGGVK**X**PHR	H3 19-40
D13	ARTKQTAR	H3 1-8
D14	ART**X**QTAR	H3 1-8
D15	QTARKSTGG	H3 5-13
D16	QTAR**X**STGG	H3 5-13
D17	STGGKAPR	H3 10-17
D18	STGG**X**APR	H3 10-17
D19	APRKQLAT	H3 15-22
D20	APR**X**QLAT	H3 15-22
D21	QLATKAAR	H3 19-26
D22	QLAT**X**AAR	H3 19-26
D23	AARKSAPA	H3 24-31
D24	AAR**X**SAPA	H3 24-31

Supplementary Figure 3: The gatekeeper E1515 of BAZ1A-BD is in proximity to the aliphatic chain of a potential acetyl-lysine ligand. (**a**) Stereo image showing electron

density for a portion of the BAZ1A bromodomain structure. The map shown is 2Fo-Fc contoured to 1σ and compared to the final structure (stick representation). **(b)** Superposition of BAZ1A-BD (green) and GCN5-BD (beige) in complex with an acetylated H4 peptide (PDB: 1E6I, [1]). (**c**) and (**d**) Sequences of peptides on arrays. Data obtained with BAZ1B-BD is used as example. Coloring was superposed onto the image to highlight peptides from the histone H4 (yellow) and H3 (blue). Residues designated by "**X**" represent the position of acetyl-lysine.

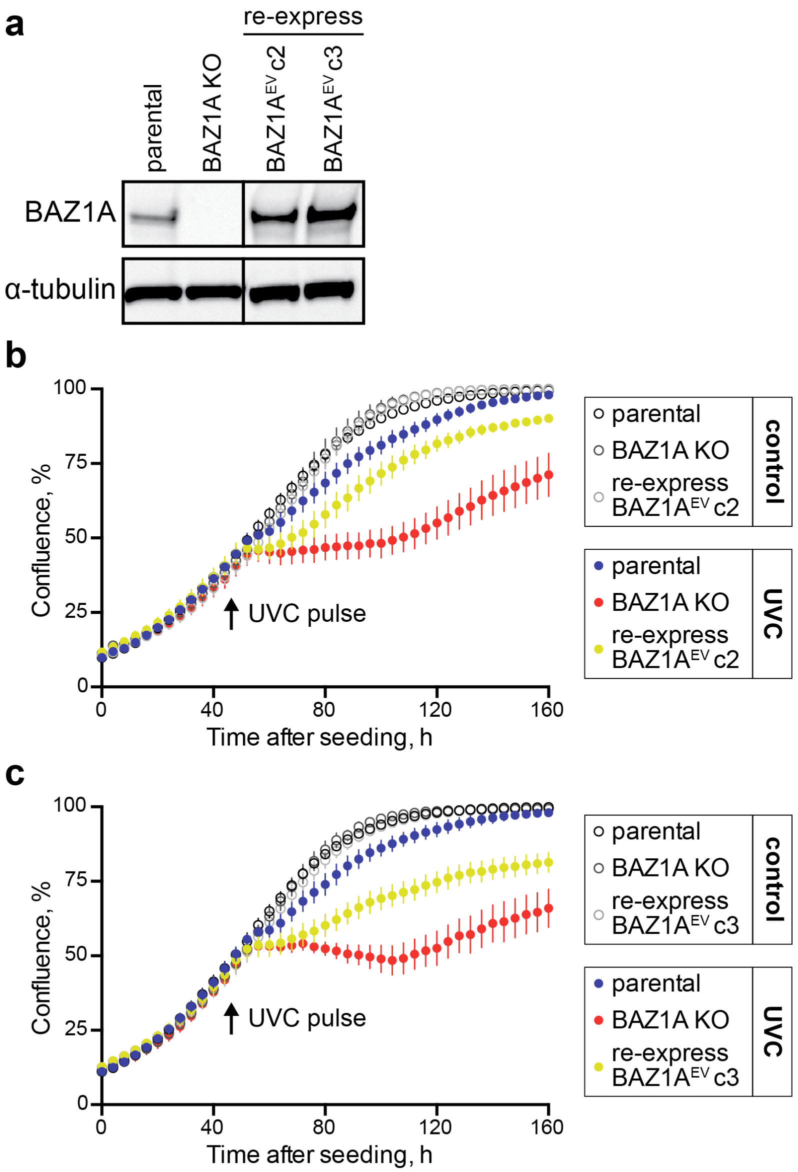

Supplementary Figure 4: A gatekeeper substitution that enhances the binding affinity of BAZ1A to acetylated peptides (BAZ1A^{EV}) causes an increase in DNA damage sensitivity (complementary to data presented in Figure 4). (**a**) Western blot analysis of BAZ1A expression in parental, *BAZ1A*-KO and two additional *BAZ1A*-KO clonal cell lines engineered to re-express BAZ1A^{EV} (E1515V) mutants (c2 and c3). (**b**) and (**c**) Confluence of the indicated cell line measured over time before and after a pulse of UVC light.

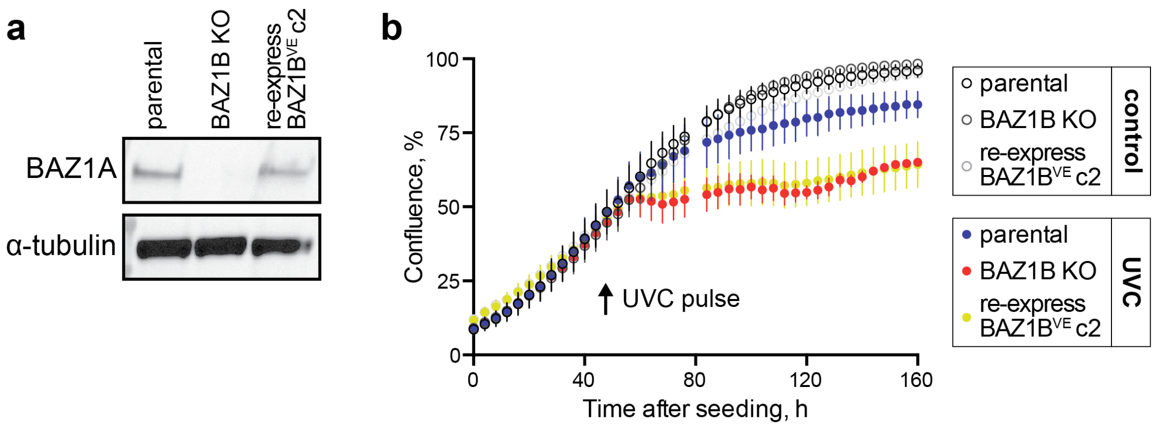

Supplementary Figure 5: A gatekeeper substitution that decreases the binding affinity of BAZ1B to acetylated peptides (BAZ1B^{VE}) causes an increase in DNA damage sensitivity (complementary to data presented in Figure 5). (**a**) Western blot analysis of BAZ1A expression in parental, *BAZ1B*-KO and an additional clonal cell line (c2) engineered to re-express a BAZ1B^{VE} (V1425E) mutant. (**b**) Confluence of the indicated cell line measured over time before and after a pulse of UVC light.

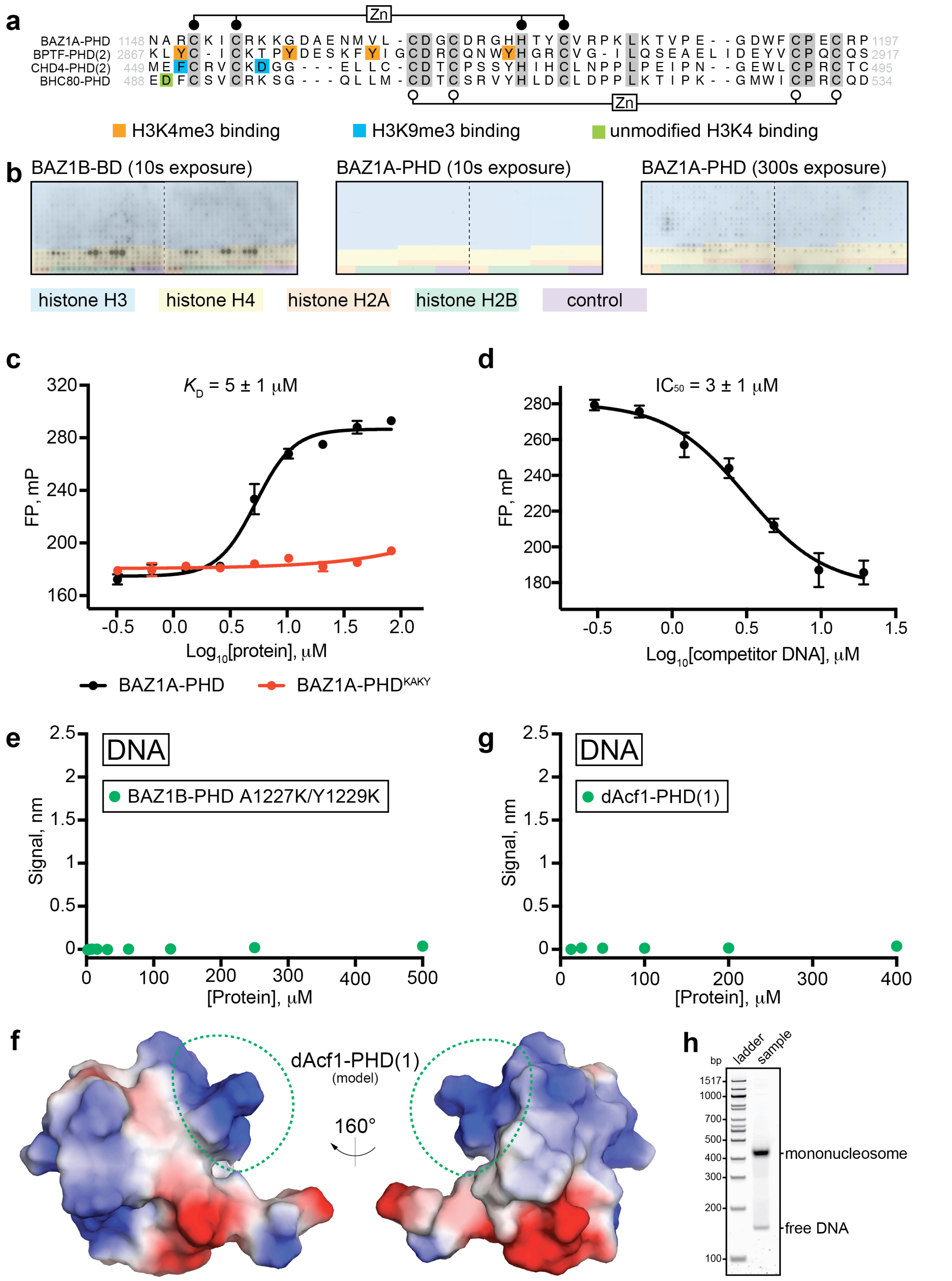

Supplementary Figure 6: The PHD module of BAZ1A fails to bind to a diverse set of modified histone peptides, but binds to DNA. (**a**) Sequence alignment of the PHD

modules of BAZ1A, BPTF, CHD4 and BHC80; amino acid boundaries are indicated. Identical residues are highlighted in grey. Residues important to bind H3K4me3, H3K9me3 and unmodified H3K4-containing peptides are highlighted in orange, cyan, and green, respectively. Filled and open circles indicate the residues of the $Cys_4HisCys_3$ motif that form the two zinc (Zn) binding sites characteristic of the PHD fold. (**b**) Representative images of MODified™ Histone Peptide Array (Active Motif) binding data for recombinant purified BAZ1A-PHD and BAZ1B-BD as positive control. Each array is spotted with 384 unique unmodified or modified histone peptides in duplicate (divided by the dotted line). Coloring was superposed onto the images to highlight peptides from the histone H3 (blue), H4 (yellow), H2A (orange), H2B (green) or control samples (purple). Weak spots observed for BAZ1A-PHD at 300 s exposure lack reproducibility and have no apparent sequence or histone mark specificity. (**c**) Fluorescence polarization upon addition of BAZ1A-PHD or the BAZ1A-PHD K1181A/K1183Y mutant (BAZ1A-PHD^{KAKY}) to 50 nM fluorescein-labeled 145 bp DNA probe. (**d**) Fluorescence polarization change for 5.3 μM BAZ1A-PHD and 50 nM fluorescein-labeled DNA probe from competition with an unlabeled DNA probe. (**e**) Steady-state biolayer interferometry measurement of DNA binding to BAZ1B-PHD A1227K/Y1229K mutant. (**f**) The structure of dAcf1-PHD(1) was modeled (Homology Modeler, MOE 2012.10, Chemical Computing Group) using as a template the crystal structure of BAZ1B-PHD (PDB: 1F62[2]). Calculated charge distribution (PyMOL 1.5.0.5., Schrodinger) on the surface of the first PHD module of *Drosophila* dAcf1 (dAcf1-PHD(1)). Blue and red indicate positively and negatively charged areas, respectively. The positively charged feature formed by K1095 and R1097 in dAcf1-PHD(1) is highlighted

with a green dotted outline. (**g**) Steady-state biolayer interferometry measurement of DNA binding to dAcf1-PHD(1). (**h**) 0.1 μg of TriDye 100 bp DNA Ladder (New England Biolabs) and 2.5 pmol of reconstituted biotinylated mononucleosomes (16-0006, EpiCypher) were separated on a native 6% DNA retardation gel, stained with SYBR® Safe (Thermo Fisher Scientific) and visualized on a Typhoon Trio (GE Healthcare Life Sciences).

a

BAZ1A-PHD peptides, residues	Relative ^{2}H uptake for uncomplexed BAZ1A-PHD, %	ARDD value, %
1-13	53	5.7
4-13	50	NA
4-15	40	4.9
14-31	26	4.2
16-31	23	NA
18-28	27	7.3
29-56	41	NA
43-55	71	15.5
56-68	58	25.1
57-68	66	NA

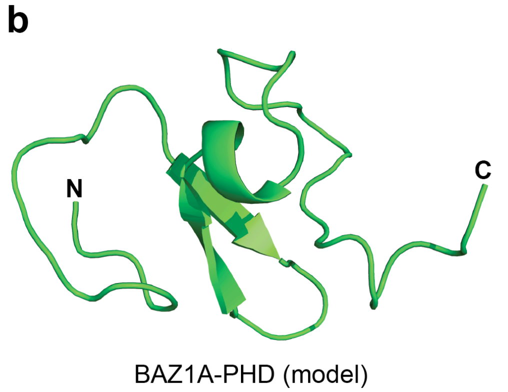

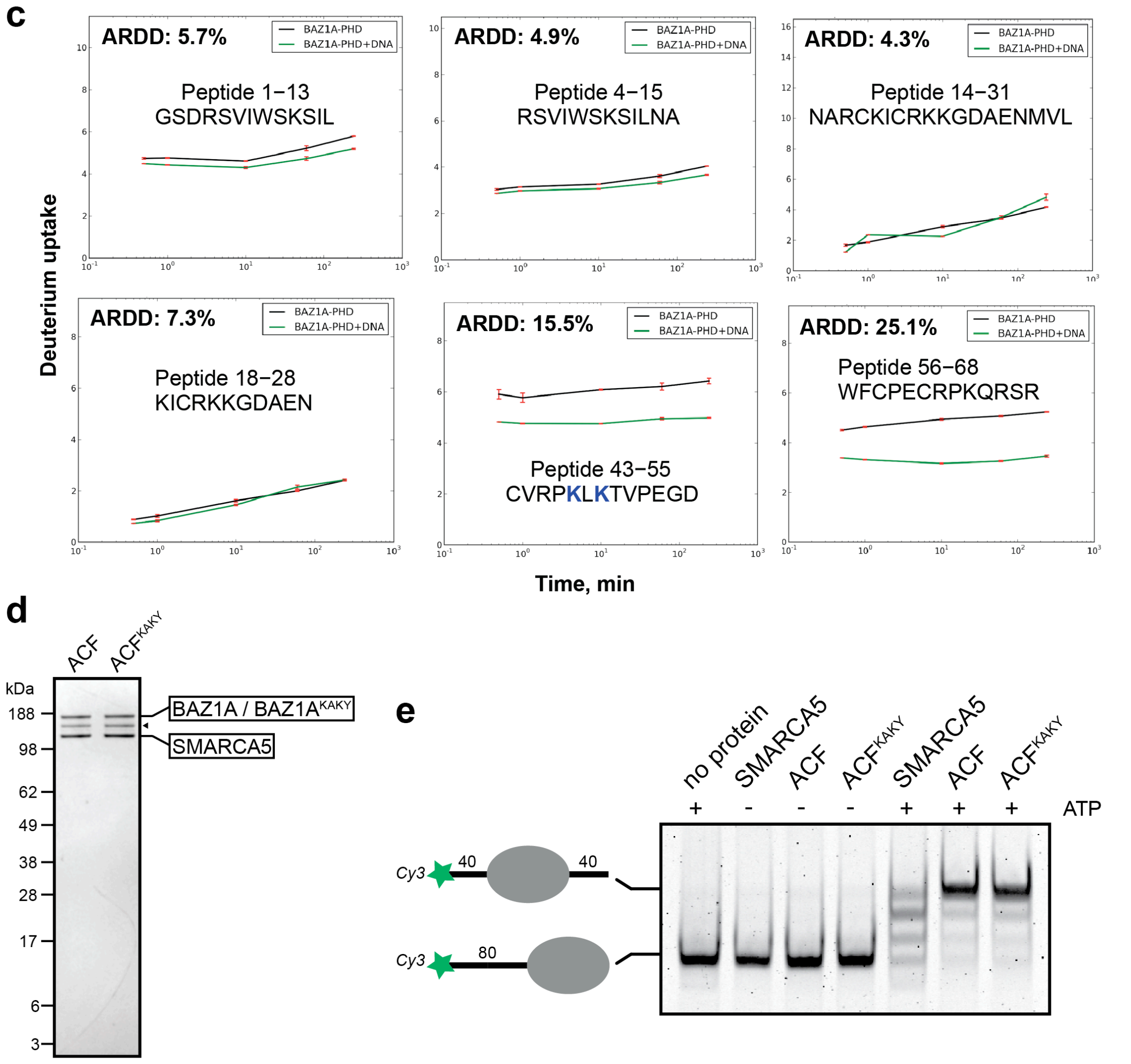

Supplementary Figure 7: The C-terminal region of BAZ1A-PHD is involved in binding DNA, but this interaction is dispensable for nucleosome mobilization. (a)

HDX-MS data table: relative deuterium (^{2}H) uptake for uncomplexed BAZ1A-PHD, and average relative deuterium uptake difference (ARDD) values across all labeling times for BAZ1A-PHD bound to DNA compared to uncomplexed BAZ1A-PHD. (**b**) Cartoon representation of the BAZ1A-PHD model highlighting predicted secondary structural features. N- and C-termini are indicated. (**c**) Time-course HDX-MS data for the six BAZ1A-PHD peptides in common between uncomplexed and DNA-bound samples. These data were used to generate the numerical values reported in **a**. (**d**) Purified ACF or ACF^{KAKY} mutant complexes. The arrowhead indicates a degradation product of BAZ1A. (**e**) Nucleosome remodeling assay using Cy3-labeled mononucleosomes. Edge-positioned mononucleosomes were incubated with the indicated protein or complex, in the absence or presence of ATP. Sliding reactions were analyzed at equilibrium. The image is representative of those from at least three independent experiments.

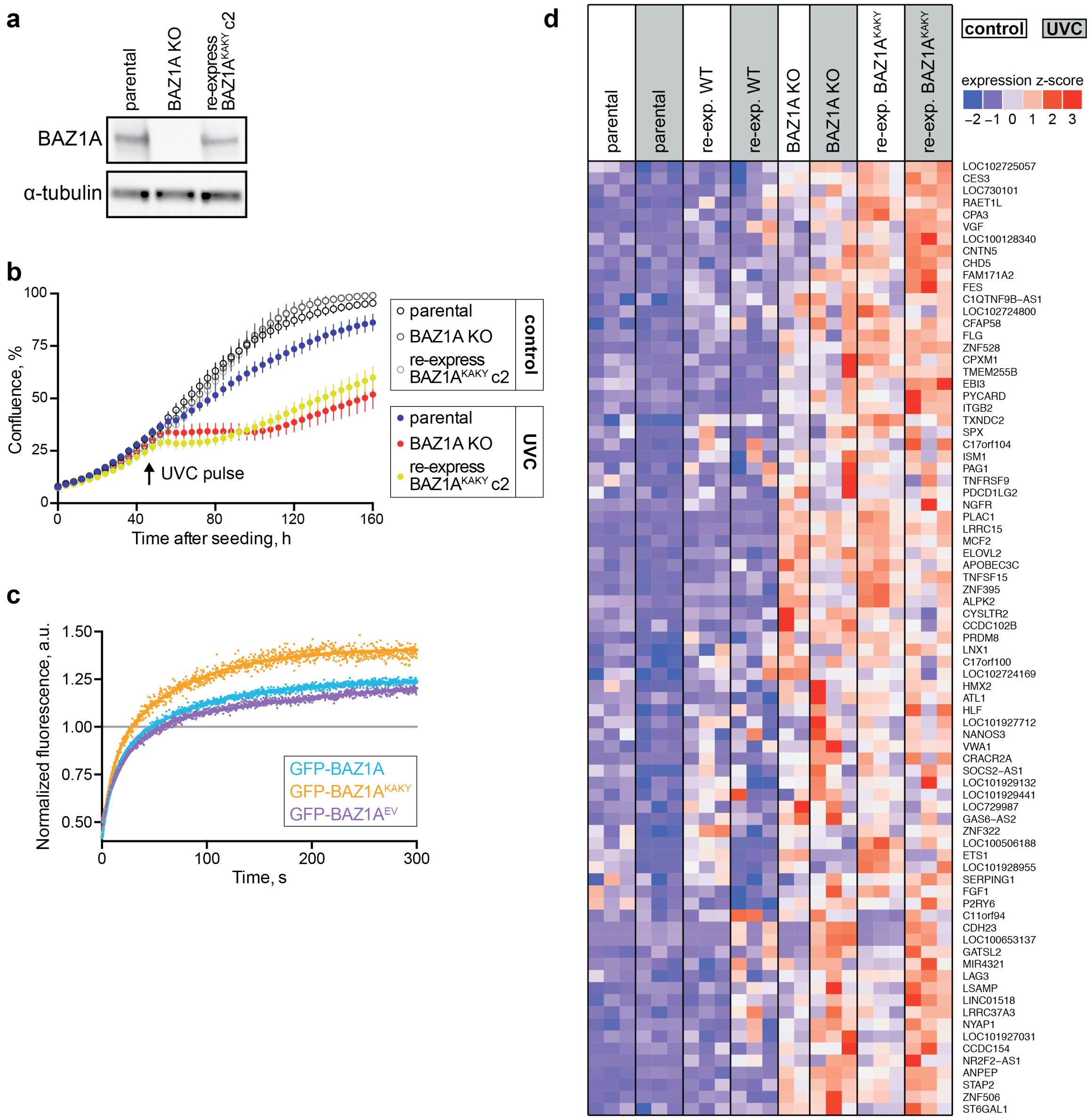

Supplementary Figure 8: Function-altering PHD substitutions are detrimental to the cellular functions of BAZ1A. (**a**) Western blot analysis of BAZ1A expression in parental, *BAZ1A*-KO and *BAZ1A*-KO cells engineered to re-express BAZ1A^{KAKY} (clone 2). (**b**) Confluence of the indicated cell line measured over time before and after a pulse of UVC light (complementary to data presented in Figure 7). (**c**) Accumulation of GFP-fusion BAZ1A proteins to sites of laser irradiation in live cells. The fluorescence signal at site of irradiation is normalized to the signal of a non-irradiated area. Signal above 1

indicates accumulation at site of damage. Numbers on the x and y axis represent the time at which the fluorescence value equals 1 and the maximum value (plateau determined by fitting), respectively. (**d**) Heat map showing the expression profiles of genes that are significantly down-regulated in parental and re-expressing BAZ1A cells after UVC treatment compared to *BAZ1A*-KO and $BAZ1A^{KAKY}$ cells. The genes that are significantly up-regulated for the same comparison are shown in Figure 7f. In both cases, only genes with the same expression profiles in parental and re-expressing BAZ1A cells, as well as *BAZ1A*-KO and $BAZ1A^{KAKY}$ cells are reported. Individual columns correspond to independent RNA extractions.

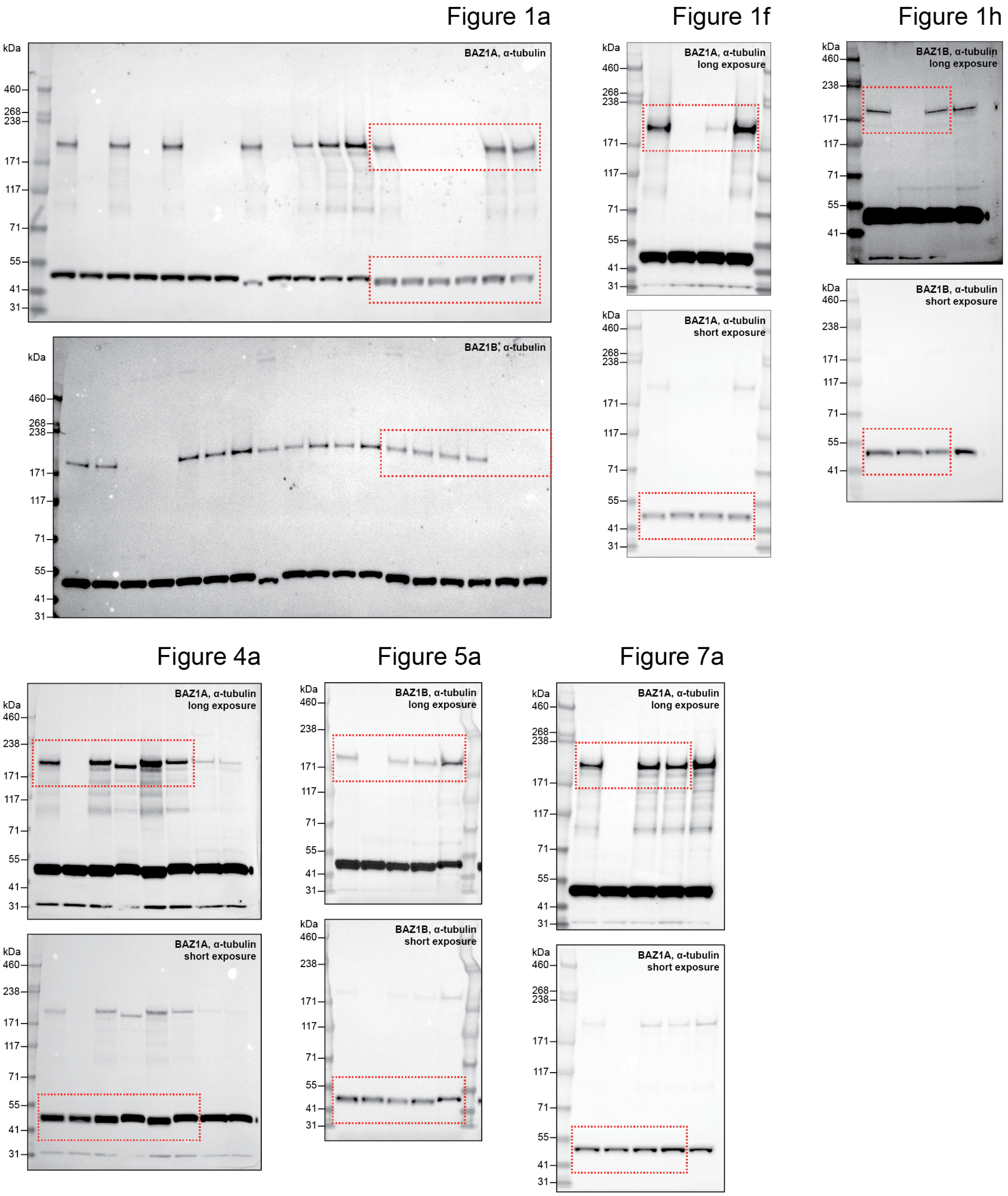

Supplementary Figure 9: Source images from which Western blot figures were extracted.

Supplementary References

1. Owen DJ*, et al*. The structural basis for the recognition of acetylated histone H4 by the bromodomain of histone acetyltransferase gcn5p. *EMBO J* **19**, 6141-6149 (2000).

2. Pascual J, Martinez-Yamout M, Dyson HJ, Wright PE. Structure of the PHD zinc finger from human Williams-Beuren syndrome transcription factor. *J Mol Biol* **304**, 723-729 (2000).
